# Determination of α-mannan content in composite yeast cultures by enzymatic hydrolysis HPLC-UV method

**DOI:** 10.3389/fnut.2025.1661855

**Published:** 2025-10-08

**Authors:** Xiaojie Zhang, Lan Yang, Haifei Lian, Zixuan Xu, Zichen Yun, Xiaohui Xu, Shixiong Liu, Qifei Luo, Majigsuren Zolzaya, Dacheng Liu

**Affiliations:** ^1^College of Veterinary Medicine, Inner Mongolia Agricultural University, Hohhot, China; ^2^Inner Mongolia Academy of Agricultural and Animal Husbandry Sciences, Inner Mongolia Agricultural and Animal Husbandry Quality Safety and Testing Research Institute (Inner Mongolia Agricultural and Animal Husbandry Science and Technology Achievement Transfer and Transformation Center), Hohhot, China; ^3^National Dairy Technology Innovation Center, Hohhot, Inner Mongolia Autonomous Region, China; ^4^Laboratory of Young Animal Physiology and Pathology, Institute of Veterinary Medicine, Mongolian University of Life Sciences, Ulaanbaatar, Mongolia

**Keywords:** composite yeast culture, α-mannan, α-mannosidase, enzymatic hydrolysis, high-performance liquid chromatography

## Abstract

**Background:**

Composite yeast culture (CYC) serves as an important functional feed additive. The content of its active ingredient, α-mannan, directly affects product quality and efficacy. However, due to the complex matrix of CYC, establishing a highly specific and accurate quantitative method for α-mannan remains a challenge.

**Methods:**

This study aimes to establish an analytical method based on enzymatic hydrolysis combined with high-performance liquid chromatography equipped with an ultraviolet detector (HPLC-UV) for the quantification of α-mannan in CYC. The method involved the specific enzymatic hydrolysis of α-mannan in the sample using α-mannosidase, followed by the release of mannose, which was then derivatized with 1-phenyl-3-methyl-5-pyrazolone (PMP) and analyzed by HPLC-UV. The actual content of α-mannan was calculated using a formula.

**Results:**

Method validation results showed that mannose exhibited good linearity within the concentration range of 0.5–400 μg/mL, with a correlation coefficient of 0.99998. The limit of detection (LOD) and limit of quantification (LOQ) were 0.063 mg/L and 0.208 mg/L, respectively. The average recovery rate was 88.020%−94.204%, with a relative standard deviation (RSD) ranging from 0.702% to 2.259%, indicating that the method has high accuracy and good precision.

**Conclusion:**

This study successfully established an efficient and reliable HPLC-UV detection method suitable for the specific quantitative analysis of α-mannan in CYC. The method exhibited good reproducibility and operational feasibility, providing robust technical support for quality control of CYC products and related functional component research.

## 1 Introduction

Composite yeast culture (CYC) is a novel microecological preparation produced through a special fermentation process, consisting of yeast cell metabolites, denatured culture medium, and a small amount of inactivated yeast cells (1). The CYC system contains both α-mannan derived from yeast cell walls and β-mannan from plant substrates. These two types of mannan differ fundamentally in their glycosidic bond configurations (α-type and β-type) and spatial structures, leading to significant differences in their physical-chemical properties and physiological functions (2–35). It is worth noting that conventional polysaccharide analysis methods (such as the phenol-sulfate method or acid hydrolysis method) cannot distinguish between these two types of mannan, leading to severe interference in the quantitative detection of functional α-mannan in CYC. This has become a key technical bottleneck constraining product quality control and functional evaluation.

In CYC, α-mannan serves as a core functional component of the yeast cell wall, characterized by a main chain composed primarily of α-1,6 glycosidic bonds and highly branched structures (α-1,2 and α-1,3 glycosidic bonds) (2–34). This structure confers strong resistance to acid, heat, and enzymatic degradation (7, 8). Extensive research has demonstrated that α-mannan possesses multiple biological activities, including immune regulation, growth promotion, improvement of intestinal microbiota, antioxidant properties, and adsorption of mycotoxins (9–15), and has been widely applied in poultry, livestock, and aquaculture (16, 18–21). Given its importance, China's Ministry of Agriculture Announcement No. 2038 explicitly stipulates that mannan content must be listed as a mandatory labeling indicator for CYC products starting from 2013. However, its functions are closely related to structural parameters such as molecular branch degree and molecular weight distribution (4, 20), making the development of a detection method capable of achieving structure-specific quantification imperative.

Currently, commonly used mannan analysis methods each have significant limitations. The phenol-sulfate method, as a widely used colorimetric method, is favored for its simplicity and lack of requirement for large-scale equipment (22). However, its accuracy and reproducibility are easily influenced by laboratory conditions and color reaction parameters, and it measures total reducing sugar content, resulting in weak specificity (22). Enzymatic methods are primarily used for the detection and analysis of plant-derived β-mannan, involving the synergistic action of enzymes such as α-galactosidase, β-mannanase, β-mannosidase, and other enzymes to hydrolyse and analyse the sample (23–28). Although its specificity and selectivity are recognized, its use of multiple expensive enzymes limits its widespread application, and there is limited research on enzymatic methods for α-mannan in yeast, with suitable enzymes yet to be further developed (23–25). High-performance liquid chromatography (HPLC) employs acid hydrolysis and HPLC coupling technology, where mannan is hydrolysed into mannose using acid hydrolysis, followed by derivatization and detection via HPLC-ultraviolet detection (UV) (29). This method offers high speed, efficiency, sensitivity, and broad applicability. However, when applied to CYC, it simultaneously hydrolyses α-mannan and β-mannan, resulting in certain inaccuracies in quantification and relatively low specificity (30).

To address the aforementioned issues, this study proposes a specific quantitative method based on enzymatic hydrolysis combined with HPLC-UV (EH-HPLC-UV) technology, drawing on previous research and existing techniques. This method employs α-mannosidase derived from jack beans, which specifically hydrolyses the glycosidic bonds in α-mannan, generating mannose (31–34, 36, 37). The mannose is then derivatized with 1-phenyl-3-methyl-5-pyrazolone (PMP) and quantified via HPLC-UV analysis. This method effectively overcomes the cross-interference of β-mannan in the complex matrix of CYC, providing a reliable solution for the accurate and specific determination of α-mannan in CYC products. It holds significant importance for enhancing product quality control and supporting precision nutrition applications.

## 2 Materials and methods

### 2.1 Preparation of composite yeast culture (CYC)

The CYC used in this study was independently developed and prepared by our laboratory. The specific process is as follows (38, 39):

Strain sources and activation: Two yeast strains—*Saccharomyces cerevisiae* (strain number BC) and *Kluyveromyces marxianus* (strain number XR4)—were selected from the collection maintained by the Microbial Preparations Research Group at the College of Veterinary Medicine, Inner Mongolia Agricultural University. The strains were first activated in malt extract agar medium and then transferred to liquid medium for propagation. The composition of the liquid medium is as follows: anhydrous glucose, molasses, yeast extract, (NH4)_2_SO4, KH_2_PO4, MgSO4, and CaCl_2_.

Solid fermentation medium preparation: The fermentation base medium was composed of the following agricultural by-products (all purchased from local markets) by mass percentage: 20% bran, 12% spray-dried corn husks, 10% corn flour, 10% rice bran, 10% cotton seed meal, 28% corn germ meal, and 10% soybean meal.

Fermentation process: The activated BC and XR4 strains were mixed in a 1:1 ratio and inoculated into the solid fermentation medium at an inoculum rate of 8% (v/w). The moisture content of the material was adjusted to 40% (w/w) by adding sterile water. Fermentation was conducted in a factory workshop using a pile fermentation method (pile height 70–80 cm), with the temperature controlled at 30 °C. During fermentation, the internal temperature of the substrate is recorded every 2 h. When fermentation reaches 24 h and the core temperature reaches 40 °C, the material is turned to ensure uniform fermentation, followed by continued fermentation until 72 h.

Post-processing: After fermentation, the product is dried at a low temperature of 45–50 °C, then ground and packaged to obtain the final CYC product.

### 2.2 Preparation of main reagents and materials

The derivatization reagent PMP-methanol solution (0.5 mol/L) was prepared freshly before use: exactly 0.4355 g of PMP (Shanghai Aladdin Biochemical Technology Co., Ltd.) was weighed and dissolved in 5 mL of methanol by vortex mixing until complete dissolution. The α-mannosidase [1 mg, derived from jack bean, purchased from Sigma-Aldrich (Shanghai) Trading Co., Ltd.] was originally supplied as a suspension in a solution containing 3.0 M (NH4)_2_SO4 and 0.1 mM zinc acetate at pH = 7.5. Prior to use, the enzyme suspension was diluted with 1 mL of ultrapure water and mixed thoroughly. The diluted solution, hereafter referred to as Enzyme A, was stored at 4 °C and used within 1 week. The yeast mannan reference standard was prepared in the laboratory: approximately 80 mg of the blank group sample (i.e., the solid-state fermented CYC medium prepared by our laboratory) was accurately weighed, and about 10 mg of yeast mannan standard [purity ≥ 99.9%, yeast-derived α-mannan, purchased from Sigma-Aldrich (Shanghai) Trading Co., Ltd.] was added. The mixture was homogenized to obtain a reference material with a known purity of approximately 11%. A total of 14 yeast culture analog samples were commercial products obtained from different manufacturers.

### 2.3 Experimental methods

#### 2.3.1 Sample pre-treatment

All samples to be tested and yeast mannan reference standards of known purity were crushed and passed through a 60-mesh sieve, then set aside for later use.

#### 2.3.2 Sample hydrolysis

Accurately weighed portions of 50–120 mg of the pretreated test sample and yeast mannan reference standard were placed into test tubes, respectively. To each tube, 2.0 mL of pre-chilled 12 M sulfuric acid solution was added. The mixtures were vortex-mixed and then reacted in an ice-water bath for 30 min. Subsequently, 10 mL of ultrapure water was added, and after mixing, the reaction was continued in a boiling water bath for another 30 min. The hydrolyzed sample solution was quantitatively transferred using a wash bottle rinsed with 50 mM MES buffer (pH 5.5) into a 50 mL centrifuge tube. Similarly, the hydrolyzed reference solution was transferred into a 100 mL conical flask. Then, 6 mL of 8.0 M sodium hydroxide solution was added to each for preliminary neutralization. Subsequently, the sample hydrolysate was brought to near 50 mL with 50 mM MES buffer (pH 5.5), and the reference hydrolysate was brought to near 100 mL. The pH was carefully adjusted to the range of 4.5–5.5 using 8.0 M sodium hydroxide solution, and each solution was finally made up to volume. A volume of 1–2 mL of the diluted solution was transferred into a microcentrifuge tube and centrifuged at 13,000 rpm for 5 min. Then, 0.2 mL of the supernatant was taken into a 15 mL centrifuge tube, and 0.2 mL of α-mannosidase solution (Enzyme A) was added. After thorough mixing, enzymatic hydrolysis was performed in a water bath at 37 °C for 17 h. Upon completion of the enzymatic hydrolysis, PMP derivatization was carried out.

#### 2.3.3 Derivatization reaction

To the hydrolyzed solutions, 400 μL of PMP-methanol solution (0.5 M) and 400 μL of sodium hydroxide (0.3 M) were added. The mixtures were incubated in a water bath at 70 °C for 10 min. Then, 500 μL of HCl (0.3 M) was added to neutralize the reaction. The solutions were washed four times with 3 mL of chloroform. The upper aqueous phase was collected and centrifuged at 10,000 rpm for 2 min. The supernatant was filtered through a 0.22 μm syringe filter and then subjected to HPLC-UV analysis.

### 2.4 Analytical conditions for HPLC-UV

All reagents used were of chromatographic grade. An HPLC-UV system (Agilent 1260, USA) equipped with an autosampler, a column thermostat, and a variable wavelength UV detector was employed. Separation of monosaccharide-PMP derivatives was achieved using an Acclaim™ C30 column (5 μm, 4.6 × 250 mm; Thermo Fisher Scientific, China). The mobile phase consisted of 0.1 mol/L ammonium acetate solution (pH 5.5) and acetonitrile in a volume ratio of 75:25, delivered at a flow rate of 1.0 mL/min. The column temperature was maintained at 35 °C, and the injection volume was 10 μL. UV detection was performed at a wavelength of 254 nm.

### 2.5 Formula for calculating the content of α-mannan

The content of α-mannan in the test sample was calculated according to Equation 1 (29, 30, 40). In polysaccharide hydrolysis analysis, a polysaccharide reference standard is introduced to obtain a correction factor (F), which compensates for inevitable losses and degradation during the experimental process, thereby yielding data that more accurately reflect the true content (41). Therefore, in this experiment, F was incorporated into the calculation of the α-mannan content. The F was calculated as described in Equation 2. The content of α-mannan in the yeast mannan reference standard was determined according to Equation 3 and expressed as a mass percentage (%).


(1) $$\begin{array}{l} X_{i}=\frac{c_{1}\times V_{1}\times n}{m_{1}}\times 0.9\times F\times 10^{-4} \end{array}$$

(2) $$\begin{array}{l} F=\frac{P\left(100\%-W\right)}{X_{i-control}} \end{array}$$

(3) $$\begin{array}{l} X_{i-control}=\frac{c_{2}\times V_{2}\times n}{m_{2}}\times 0.9\times 10^{-4} \end{array}$$

In the formulas: *Xi* represents the α-mannan content in the test sample (%). *Xi-control* denotes the α-mannan content in the yeast mannan control (%). The mannose concentration in the test sample hydrolysate and the yeast mannan control hydrolysate are designated as *c*_1_ (μg/mL) and *c*_2_ (μg/mL), respectively. *V*_1_ and *V*_2_ correspond to the final volumes (mL) of the test sample and control hydrolysates. The dilution factor of the hydrolysate is represented by n (*n* = 8.5), and 0.9 is the conversion factor from mannose to α-mannan. The mass of the test sample and yeast mannan control are indicated as *m*_1_ (g) and *m*_2_ (g), respectively. Additionally, *F* is the correction factor accounting for α-mannan loss during the analytical procedure, *P* refers to the purity (%) of the yeast mannan control, and *W* represents its moisture content (%). All measurement results are expressed as the arithmetic mean ± standard deviation of parallel measurements, with values retained to three significant figures.

### 2.6 Methodological validation

The established detection method was validated through linearity, precision, repeatability, and spiked recovery tests.

Establishment of the mannitol standard curve: An appropriate amount of mannose standard (Shanghai Aladdin Biochemical Technology Co., Ltd.) was accurately weighed and dissolved in ultrapure water to prepare a stock solution with a concentration of 1.0 mg/mL, which was stored at 4 °C for further use. A series of working standard solutions at concentrations of 0.5, 5, 10, 50, 100, and 400 μg/mL were prepared by serial dilution of the stock solution. All diluted solutions were prepared freshly before use. The standard working solutions were subjected to PMP derivatization as described above. The reaction mixtures were filtered through a 0.22 μm membrane and analyzed by HPLC-UV. A standard curve was constructed by plotting the concentration of mannose (x, μg/mL) against the corresponding peak area (y), and a regression equation was fitted using Excel.

Precision test: Mannose standard working solutions at three concentrations (5, 50, and 100 μg/mL) were selected for the precision test. Each solution was analyzed six times under the specified chromatographic conditions. The average peak area and relative standard deviation (RSD) were calculated.

Repeatability test: The repeatability test was performed using the laboratory-prepared CYC as the test sample. Six replicates of the test sample were processed following the above experimental procedures. Each replicate was analyzed under the same chromatographic conditions, and the average peak area along with the RSD was computed.

Spike recovery test: The spike recovery test was conducted by adding three different amounts of mannose standard to the laboratory-prepared CYC sample. Each spiking level was repeated six times. The samples were processed according to the described method and analyzed under the specified chromatographic conditions. The average peak area and RSD were determined to evaluate the recovery rate.

### 2.7 Application of the detection method

The established detection method was applied to analyze 14 commercially available yeast culture analog samples (Market Samples) and the CYC sample prepared in our laboratory (test group). Each sample was independently measured in triplicate, and the results are expressed as arithmetic mean ± standard deviation.

### 2.8 Statistical analysis

All experimental data were recorded on the day of measurement. Data organization and preliminary calculations were performed using Microsoft Excel 2021. Statistical analysis was carried out using GraphPad Prism version 9.5.1. One-way analysis of variance (ANOVA) was applied, followed by Tukey's *post-hoc* test for multiple comparisons. The results are presented as mean ± standard deviation, and a *P*-*value* of less than 0.05 was considered statistically significant.

## 3 Results

### 3.1 Optimization of enzymatic hydrolysis duration

**Figure 1:**
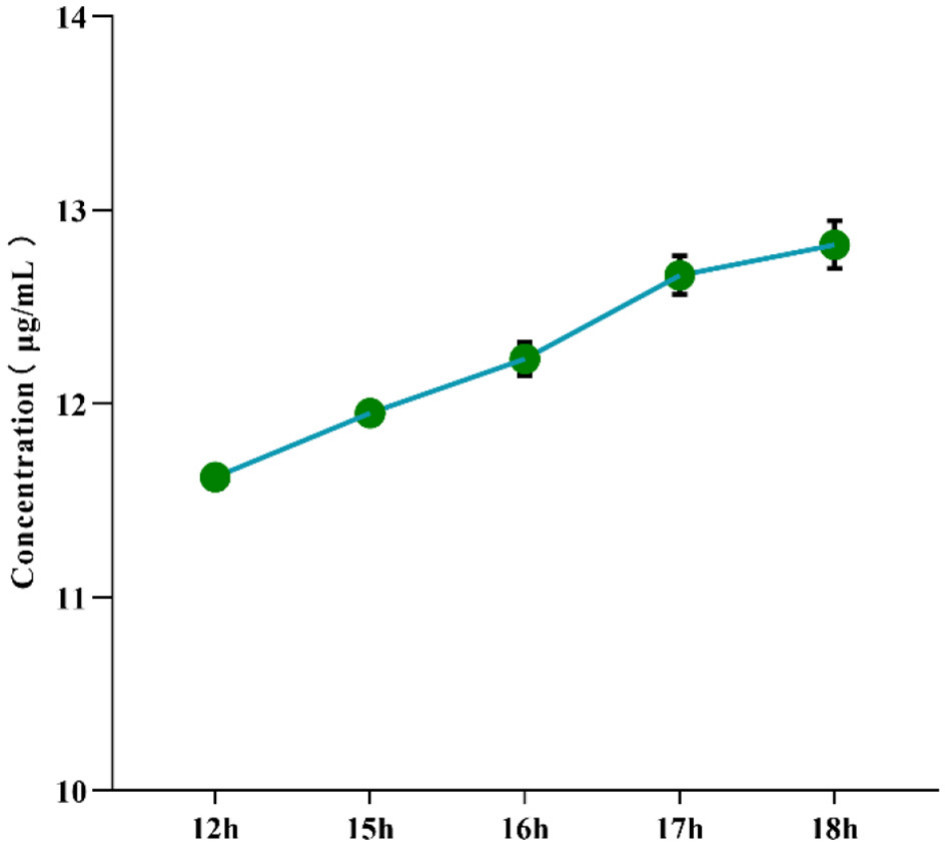
Effect of enzymatic hydrolysis time on the concentration of mannose derivatives in yeast mannan reference substance controls.

To determine the optimal hydrolysis time for α-mannosidase, the effect of different enzymatic hydrolysis durations (12–18 h) on the concentration of mannose derivatives in the yeast mannan reference standard was investigated under fixed reaction conditions. As shown in Figure 1, the concentration of mannose derivatives gradually increased with prolonged hydrolysis time. When the hydrolysis time reached 17 h, the concentration of mannose derivatives was 12.664 ± 0.101 μg/mL (*P* < 0.001), with an RSD of 0.795% and a degree of hydrolysis (DH) of 94.700%, indicating high stability of the reaction system. Extending the hydrolysis time to 18 h resulted in a concentration of 12.822 ± 0.124 μg/mL and a DH of 94.659%, which were not significantly different from those of the 17 h group (*P* > 0.05); however, the RSD increased slightly to 0.971%. These results demonstrate that a DH of 94.700% was achieved at 17 h, indicating sufficient hydrolysis of α-mannan. Further extension of the reaction time did not significantly improve hydrolysis efficiency. Moreover, the RSD at 17 h remained below 0.8%, reflecting excellent repeatability. Therefore, 17 h was determined to be the optimal enzymatic hydrolysis time for α-mannosidase.

### 3.2 Selection of derivative duration and extraction frequency

**Figure 2:**
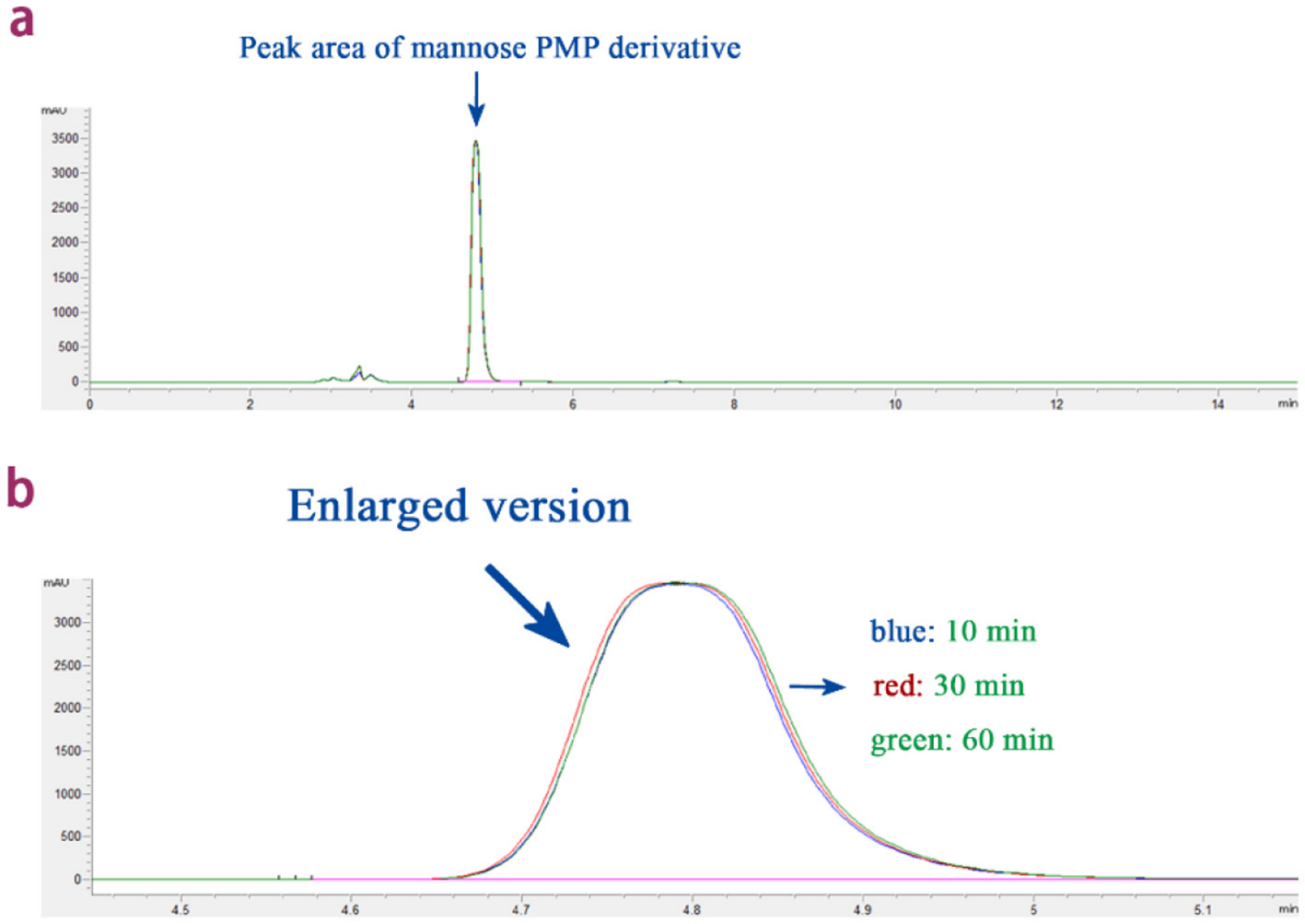
The effect of derivatization reaction time on the peak area of mannose derivatives. **(a)** Overlapping plots of peak areas of mannose derivatives under different derivatization reaction times. **(b)** Enlarged version: overlap plot of peak areas of mannose derivatives at different derivatization reaction times.

Under the premise of unchanged other conditions, this experiment tested the effects of different derivatization reaction times (10, 30, and 60 min) on the concentration of mannose derivatives. The results, as shown in Figure 2, indicate that the chromatographic peaks of the mannose derivatives for 10, 30, and 60 min were nearly overlapping. Multiple comparison analysis of all group data revealed that prolonging the derivatization reaction time did not significantly increase the concentration of mannose derivatives (*P* > 0.05). Therefore, considering the improvement in reaction efficiency and the reduction in time cost, 10 min was determined to be the optimal derivatization reaction time for this experiment.

**Figure 3:**
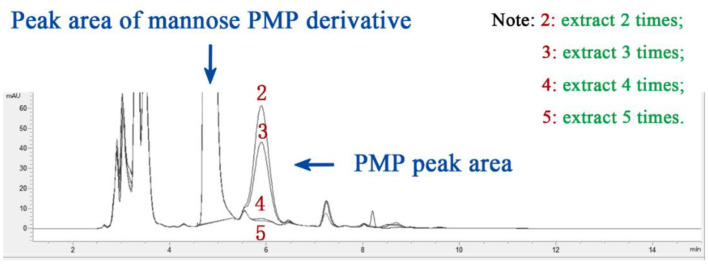
The effect of extraction times on PMP peak area.

In this experiment, chloroform was selected as the extraction solvent (42), with a single dosage of 3 mL. The influence of different numbers of extraction cycles (2, 3, 4, and 5 times) on the PMP peak area was tested. The results (Figure 3) showed that the PMP peak area gradually decreased as the number of extractions increased. When the number of extractions exceeded four, the PMP peak area was almost reduced to zero. Taking into consideration both extraction efficiency and experimental practicality, four extraction cycles were determined to be the optimal number for this experiment.

### 3.3 Methodological validation

#### 3.3.1 Linear relationship

**Figure 4:**
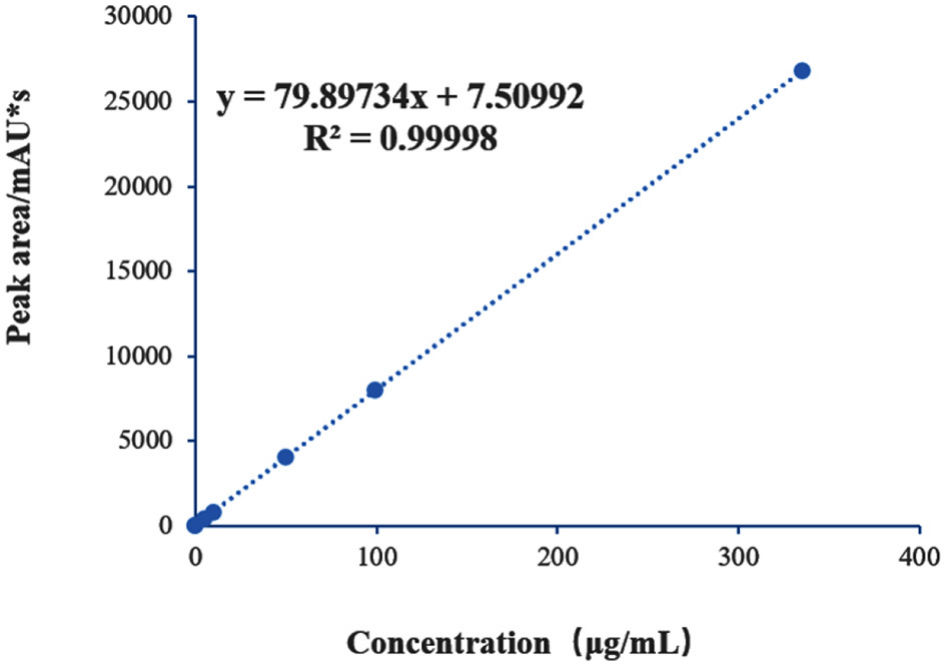
Standard curve of mannose.

A standard curve for mannose was established (Figure 4), showing a good linear relationship between mannose concentration and chromatographic peak area within the range of 0.5–400 μg/mL. The fitted regression equation was y = 79.89734x + 7.50992, with *R*^2^ = 0.99998. The limit of detection (LOD) and limit of quantification (LOQ), calculated based on signal-to-noise ratios (S/N) of 3 and 10, were 0.063 mg/L and 0.208 mg/L, respectively. These results demonstrate that the method exhibits high precision and accuracy, supporting its suitability as a reliable approach for quantitative analysis.

#### 3.3.2 The results of method precision and sample repeatability tests

The results of the method precision test are presented in Table 1. The RSD values were all below 1%, indicating excellent analytical performance of the instrument under the current chromatographic conditions.

**Table 1:** Method precision test results (*n* = 6).

**Items**	**Mannose standard working solution with different concentrations**		
	**5** μ**g/mL**	**50** μ**g/mL**	**100** μ**g/mL**
Retention time/min	4.778	4.774	4.756
	4.774	4.776	4.776
	4.769	4.774	4.762
	4.794	4.768	4.753
	4.792	4.786	4.780
	4.756	4.770	4.777
Average/min	4.777	4.775	4.767
RSD/%	0.300	0.132	0.247
Peak area/mAU^*^s	280.090	4,332.427	8,898.688
	279.998	4,325.938	8,885.146
	279.602	4,328.933	8,871.964
	283.068	4,334.205	8,888.195
	284.149	4,327.173	8,877.925
	284.537	4,331.709	8,879.805
Average/mAU^*^s	281.907	4,330.064	8,883.620
RSD/%	0.802	0.075	0.105

As shown in Table 2, the repeatability test results demonstrated minimal variation in the chromatographic peak areas across multiple measurements of the same sample, with all RSD values also below 1%, suggesting negligible random error in the experiment. Therefore, the proposed method exhibits high reliability and validity.

**Table 2:** The results of method repeatability test (*n* = 6).

**Items**	**Retention time/min**	Peak area/mAU^*^s	Average/mAU^*^s	**RSD/%**
Sample of mannan	4.721	113.982	113.964	0.155
	4.737	114.262		
	4.741	113.843		
	4.743	114.049		
	4.752	113.776		
	4.749	113.871		

#### 3.3.3 Results of spiked recovery rate test

The experimental results were summarized, and the spike recovery rates were calculated (Table 3). Over the spiking range of 0.2–0.8 mg, the average recovery rates ranged from 88.020% to 94.204%, with RSD values between 0.702% and 2.259%. These results demonstrate that the method possesses high applicability and feasibility, and is capable of meeting the requirements for routine detection.

**Table 3:** Results of spiked recovery test (*n* = 6).

**Items**	**Mannose**		
Initial value/mg	0.529 ± 0.004		
Additive amount/mg	0.2	0.4	0.8
	0.713	0.897	1.251
	0.712	0.896	1.254
Measured value/mg	0.712	0.895	1.258
	0.713	0.896	1.256
	0.714	0.896	1.255
	0.717	0.897	1.256
	88.020	90.058	89.347
	91.639	91.920	90.747
Spiked recovery/%	92.285	92.065	91.314
	92.574	92.146	91.019
	92.617	92.032	90.879
	94.204	92.223	90.908
Average value/%	91.890	91.741	90.702
RSD/%	2.259	0.905	0.762

### 3.4 Comparison between EH-HPLC-UV and HPLC methods

Under optimized conditions, the yeast mannan reference standard was analyzed using both the EH-HPLC-UV and HPLC methods. As shown in Table 4, the correction factor (F) obtained by the EH-HPLC-UV method was 1.113 (RSD = 3.978%), while that by the HPLC method was 1.507 (RSD = 3.095%). The result derived from the EH-HPLC-UV method was closer to the theoretical value, and its F-value was nearer to 1, indicating that this method offers higher accuracy and reliability, making it more suitable for the quantitative analysis of yeast mannan.

**Table 4:** Determination of yeast mannan reference by different assay methods (*n* = 6).

**Items**		**Yeast mannan reference standard**						**Average value/%**	**RSD/%**
EH-HPLC-UV	Theoretical content/%	11.789	11.552	11.684	11.751	11.448	11.321		
	Measured content/%	10.015	10.512	10.267	10.730	10.211	10.820	10.424	3.035
	F	1.177	1.099	1.138	1.095	1.121	1.046	1.113	3.978
HPLC	Theoretical content/%	11.670	11.308	11.958	11.512	11.422	12.100		
	Measured content/%	7.430	7.675	7.841	7.461	7.696	8.354	7.743	4.350
	F	1.571	1.473	1.525	1.543	1.484	1.448	1.507	3.095

F, The correction factor.

### 3.5 Application of EH-HPLC-UV detection method

The α-mannan content in 14 market samples (Market Samples) and the laboratory-prepared CYC sample (Test group) was determined using the EH-HPLC-UV method. The results (Table 5) show that α-mannan was detected in all samples, with levels varying substantially. With the exception of sample 11, all products labeled as containing ≥2.0% or ≥25% α-mannan failed to meet their claimed values. The majority of samples had α-mannan levels in the range of 0.5–1.5%, while samples 10 and 12 exhibited significantly higher contents, suggesting possible process intensification or external addition. Furthermore, 78.6% of the samples showed an RSD above 2.0%, indicating that the complex sample matrices may affect the reproducibility of the detection.

**Table 5:** Detection results of α-mannan in different yeast cultures (%).

**Items**	**Content of mannan**	**α-Mannan content**	**RSD**
	**Labeled content**	**Measured content**	
Test group	–	0.553 ± 0.014	2.452
Market samples 1	–	0.556 ± 0.017	3.061
Market samples 2	≥2.0	0.765 ± 0.004	0.539
Market samples 3	≥2.0	0.579 ± 0.019	3.231
Market samples 4	≥2.0	0.709 ± 0.033	4.700
Market samples 5	–	0.598 ± 0.024	4.073
Market samples 6	–	0.328 ± 0.016	4.837
Market samples 7	–	1.47 ± 0.041	2.772
Market samples 8	–	0.618 ± 0.028	4.508
Market samples 9	≥2.0	0.932 ± 0.035	3.750
Market samples 10	≥25	10.862 ± 0.323	2.977
Market samples 11	≥0.3	0.389 ± 0.014	3.618
Market samples 12	–	3.256 ± 0.035	1.067
Market samples 13	–	1.137 ± 0.034	2.947
Market samples 14	–	0.488 ± 0.008	1.626

–, Not labeled.

## 4 Discussion

In plants, mannan is predominantly present as β-mannan, a key component of the hemicellulose family. Its core structure consists of a backbone of mannose residues linked by β-1,4-glycosidic bonds, which may be accompanied by combinations of glucose and mannose residues, as well as side chains of α-1,6-linked galactose residues (5, 6). β-mannan participates in cell wall metabolism and plays a critical role in plant growth, maturation, and senescence. It is often enriched in the endosperm of leguminous plants and is commonly found in various cereals that form the basis of livestock feed. However, due to its potential interference with nutrient digestion and utilization, it is considered an anti-nutritional factor (43, 44). In contrast, the mannan found in yeast is α-mannan, which is fundamentally distinct from plant-derived β-mannan. It is a biologically active polysaccharide with immunostimulatory properties and is regarded as an important nutrient (9–15). CYC is produced through the fermentation of agricultural by-products such as corn and soybean meal using yeast strains. As a result, CYC contains both α-mannan and β-mannan. Specific detection of α-mannan in CYC thus requires a specialized enzymatic hydrolysis strategy. Previous studies have established methods for the detection and analysis of plant-derived β-mannan using synergistic enzyme systems including β-mannanase, β-mannosidase, and accessory enzymes (24–28). However, there remains a gap in enzymatic strategies specifically targeting yeast-derived α-mannan. The α-mannosidase used in this study is an acid hydrolase belonging to the Glycoside Hydrolase Family GH 38 according to the CAZy database (http://www.cazy.org/). It is an exoglycosidase capable of hydrolyzing α-1,2-, α-1,3-, and α-1,6-glycosidic linkages, releasing mannose from various synthetic and natural α-mannosides. Purified forms of this enzyme have been widely employed to determine mannose linkages in glycoproteins (31–34, 36, 37). We found that α-mannosidase alone—without the requirement for exogenous accessory enzymes—was able to hydrolyze both the main chain and side chains of α-mannan, resulting in the release of mannose. This observation is consistent with findings reported by Dohi et al. (33) and Liu et al. (45) respective studies.

In the experiment to determine the optimal enzymatic hydrolysis duration, this study identified 17 h as the optimum, which is consistent with the 16 h hydrolysis period reported by Liu et al. (45) for mannan hydrolysis, but differs considerably from the 1–8 h reported by Dohi et al. (33). Several factors may account for this discrepancy: (1) The mannan substrates examined in these studies differ in source, configuration, structure, and molecular weight. Even when hydrolyzed with the same jack bean α-mannosidase, the efficacy and efficiency of hydrolysis may vary. (2) Differences in substrate concentrations across studies may lead to substrate inhibition effects, as described by the Michaelis-Menten equation, altering enzymatic kinetic parameters (such as Km and Vmax) and consequently affecting the required hydrolysis time (46–48). (3) Variations in the cleavage specificity and pathways of jack bean α-mannosidase also contribute. This enzyme exhibits broad specificity toward α-1,2-, α-1,3-, and α-1,6-mannosidic linkages rather than targeting a single bond type, resulting in relatively low working specificity and somewhat stochastic hydrolysis sites. As noted by Dohi and Kołaczkowski, the recognition and cleavage order of specific sites in mannans with different molecular weights and branching structures vary significantly with α-mannosidase. Differential steric hindrance during sequential cleavage may further influence overall hydrolysis efficiency and duration (33, 49). Future studies could explore the effects of varying substrate concentrations and evaluate the synergistic use of α-mannosidases from families GH92 and GH99—which specifically target α-1,2/α-1,3 linkages—in combination with jack bean α-mannosidase, with the aim of improving hydrolysis efficiency and reducing incubation time.

For monosaccharide analysis, this study employed the PMP derivatization followed by HPLC-UV analysis, achieving high-sensitivity detection of mannose (50). The derivatization reaction of mannose was observed to reach equilibrium within 10 min. In contrast, a separate experiment within this study focusing on the derivatization time of glucose revealed that 60 min were required for complete derivatization, which differs from the 30 min derivatization period reported by Fan B for monosaccharide-PMP derivatization (51). This discrepancy may largely be attributed to differences in the monosaccharide content within the derived samples. The current method was optimized specifically for a single target analyte—mannose—present at low concentration, which significantly shortened the analytical cycle and improved detection efficiency. Therefore, it is necessary to adjust the derivatization time according to the specific research context, particularly considering the concentration of the target analyte. When such information is unavailable, conducting preliminary tests to determine the optimal derivatization duration is essential.

In polysaccharide hydrolysis analysis, strong acid hydrolysis is widely used due to its high efficiency and simplicity (22, 29). However, strong acids can promote the conversion of monosaccharides into furan compounds (e.g., furfural and hydroxymethylfurfural), thereby interfering with content analysis (30). Therefore, it is necessary to introduce polysaccharide reference standards to derive correction factors that compensate for inevitable hydrolysis losses and degradation. A correction factor closer to 1 indicates higher accuracy.

This study used yeast mannan reference standard for calibration and evaluated the effects of EH-HPLC-UV and HPLC methods on the quantification of yeast mannan reference standard. The results showed that the correction factor obtained by the EH-HPLC-UV method (mean *F* = 1.113) was closer to 1, indicating better controllability of the enzymatic system in hydrolyzing glycan chains and demonstrating that the yeast mannan reference effectively corrected for hydrolysis losses. Furthermore, the range of F-values observed in this study (1.046–1.177) partially overlapped with the range (0.86–1.18) reported by Zhou et al. (41), confirming the necessity of correction in experimental procedures and supporting the reliability of the method used in this study. It is noteworthy that at the initial stage of the experiment, reference standards should be included in each run to ensure immediate correction. Once the experimental procedure is well-established and operator technique has stabilized, the correction factor may be updated on a monthly basis.

The EH-HPLC-UV method established in this study exhibits distinctly different characteristics from the traditional HPLC method for the quantification of α-mannan. Although the conventional HPLC method, which relies on acid hydrolysis to release monosaccharides, is operationally simpler and more cost-effective, its major limitation lies in the inability to distinguish between α- and β-configurational mannans. In complex samples such as composite yeast cultures that contain plant-derived β-mannan, this method is prone to yield deviations from the true values. In contrast, the EH-HPLC-UV method employs α-mannosidase for specific enzymatic hydrolysis of α-mannan. Despite requiring a 17 h hydrolysis process and dependence on relatively expensive enzyme reagents, it significantly improves the accuracy and specificity of quantification of the target component. It is particularly suitable for complex sample matrices and demonstrates superior anti-interference capability and structural recognition in practical samples with significant matrix effects. Nevertheless, the proposed method also has notable limitations, including prolonged pretreatment time, multiple operational steps, and reliance on specific enzymes, which may restrict its application in high-throughput routine detection. Therefore, this method is more appropriate for scenarios requiring high accuracy, complex sample matrices, or specific identification of α-mannan—such as functional ingredient verification, product quality control, and standardization. In future studies, efforts can be directed toward optimizing enzymatic hydrolysis efficiency, developing lower-cost enzyme alternatives, and integrating or automating procedural steps to enhance the method's operational convenience and economic feasibility, thereby expanding its potential for broader application in practical detection workflows.

MES buffer solution, a zwitterionic buffer, is effective within the pH range of 5.5–7.0. It exhibits good buffering capacity, low ionic strength, absence of metal chelation effects, and does not interfere with enzymatic activity (52). Due to these properties, MES buffer solution is widely used in biochemical and molecular biological experiments. In this study, considering that enzymatic activity is highly dependent on pH and that the reaction extended over a prolonged period (17 h), a buffer capable of providing stable pH control over an extended timeframe was essential to minimize fluctuations that could affect enzymatic performance (53). Therefore, after comprehensive evaluation, MES was selected. Studies by Liu et al. (45) and Dohi et al. (33) also support the suitability of MES for prolonged enzymatic reactions, providing a theoretical basis for its use in this study.

In this study, the EH-HPLC-UV method was employed to analyze 14 commercial samples of yeast culture products. The results revealed considerable variation in the α-mannan content among products from different manufacturers. The observed discrepancies can be attributed to the following factors: (1) The polysaccharide composition of yeast cell walls varies across yeast strains and cultivation conditions. In particular, mannan, as a major component of the yeast cell wall, may differ in content and structure depending on the strain. As reported by Boutros significant differences may exist in the cell wall polysaccharide profiles between *Saccharomyces cerevisiae* and other yeast strains, which affects their adaptability and functionality in various environments (54). (2) The structure and content of mannan are influenced not only by the strain but also strongly by the culture conditions. The thickness of the yeast cell wall and the composition of polysaccharides may vary in different media, directly affecting the mannan content. This observation aligns with the findings of Pereyra et al. (55), who reported cell wall thickening in yeast cultivated using agro-industrial waste materials. Furthermore, nutrient availability and environmental conditions—such as temperature and pH—also impact the synthesis and accumulation of mannan, as thoroughly demonstrated in studies by Farinha (35). (3) The biosynthetic pathways of mannan and the expression of related genes also exhibit variability among yeast strains. Differences in the expression levels of these genes may influence the efficiency of mannan synthesis and its final content. Research by Yamasaki-Yashiki et al. (56) elucidated the biosynthetic mechanisms of mannan in yeast at the genetic level, providing molecular support for the results obtained in this study. Therefore, the α-mannan content in yeast culture products is influenced by multiple factors, including the genetic background of the strain, culture conditions, and structural characteristics of the cell wall. These factors collectively contribute to the variability in mannan content across different yeast culture products. Additionally, a notable discrepancy was observed between the mannan content measured in this study and the values labeled on commercial products, with the detected values generally being lower than those claimed. This discrepancy may stem from differences in detection methods. Currently, there is no standardized detection method mandated in China, and product labels often do not specify the analytical technique used, making it difficult to evaluate the accuracy of the claimed values or assess the true α-mannan content in CYC products. Hence, it is essential to establish grading standards for α-mannan and unify detection methodologies to regulate market practices. The EH-HPLC-UV method developed in this study enables quantitative detection of α-mannan across diverse commercial samples and can serve as a novel analytical approach. It provides technical support for quality control and product development of CYC, and offers valuable data reference for future research and production.

## 5 Conclusion

In summary, this study successfully established an enzymatic hydrolysis-HPLC (EH-HPLC-UV) method for the quantitative detection of α-mannan in CYC. Although this method requires a longer pretreatment time compared to traditional HPLC, it significantly improves the accuracy and specificity of α-mannan quantification by leveraging the specific enzymatic hydrolysis of α-mannosidase, effectively avoiding cross-interference from β-mannan present in the sample. Validation demonstrated that the method performs well in terms of linear range (0.5–400 μg/mL), precision, repeatability (RSD < 1%), and spike recovery (88.020%−94.204%). Furthermore, the detection results of 14 commercial samples confirmed its reliability and stability in practical applications. Although the method has certain limitations in throughput and cost, its ability to specifically identify target active ingredients in complex matrices makes it highly suitable for quality control and functional component analysis of CYC products. This study provides a feasible and highly specific analytical method for the quality assessment of functional polysaccharides in CYC, contributing positively to the further development and standardization of related feed and functional food products.

## Data Availability

The original contributions presented in the study are included in the article/supplementary material, further inquiries can be directed to the corresponding author.
